# Expanding Use of Nurse Home Visiting for Community Psychiatric Care in Japan

**DOI:** 10.1007/s11126-020-09721-w

**Published:** 2020-02-24

**Authors:** Mami Kayama, Nozomi Setoya, Colleen Doyle

**Affiliations:** 1 Psychiatric & Mental Health Nursing, Graduate school of Nursing, St. Luke’s International University, 10-1 Akashi-cho, Chuo-ku, Tokyo, 104-0044 Japan; 2 National Ageing Research Institute, Royal Melbourne Hospital, PO Box 2127, Parkville, Victoria 3050 Australia

**Keywords:** Home visiting nursing, Community psychiatry, Policy change, Japan

## Abstract

In Japan, Community-based integrated care systems are being built in response to a super-aged society and policies of de-institutionalization. In this paper, we present findings and discussion of our review about Japanese psychiatric home visit nursing services provided by Home Visit Nursing Stations (HVNS). We have examined documents, investigated the implementation rate and summarized findings of the surveys of home visiting services from 2006 to 2016. The number of users of psychiatric home visiting services during 2007 to 2015 increased from 13,532 to 52,203. From 2013 to 2015 there was a large increase in user numbers, from 31,248 to 52,208. The implementation rate of psychiatric home visiting also increased steadily from 35.5% in 2006 to 58.3% in 2016. These changes reflected the impact of policy on psychiatric service usage in Japan. We should be able to detect the outcome of psychiatric home visiting nursing in influencing patient’s quality of daily life and their recovery.

## Background

In Japan, community-based integrated care systems are being built to accommodate a super-aged society, within economic constraints. However Japan still has the highest ratio of beds devoted to psychiatric patients, in the world (2.7 beds per 1000 persons) [1]. By the late 1990’s and early twenty-first century, Japan was implementing mental health reform including deinstitutionalization and expansion of community mental health services [2]. While expanding community care for people with mental illness remains a formal policy in most countries, the extent to which community care is offered and the programming is variable. From 2013, according to the Medical Care Act, each prefecture in Japan had to formulate a Medical Care Plan for patients with mental health problems, to ensure the medical care coordination system was applied and appropriate care was provided with good quality. This law regulated five major illnesses; Cancer, Cerebral apoplexy, Acute Myocardial infarction, Diabetes and Psychiatric Diseases.

As part of this law, the governmental guidelines recommended that home visiting nursing services were included in this plan. Home Visit Nursing Stations (HVNS) were launched in 1992 as the supplier of home visit nursing service and were further distributed by a Long-Term Care Insurance Act in 2000. The director of HVNS was intended to be a Registered Nurse and the service is paid by national medical insurance. Requests for home visits are received by referrals from both general practitioners and psychiatrists, and HVNS now provide services to users with various illnesses. Home visiting services for psychiatric patients stem from both hospital and HVNS themselves. The contents of services are slightly different. In Japan, psychiatric hospitals tend to be located some distance from cities. By contrast HVNS are close to the patient’s residence town and daily living area. For community-based integrated care systems, HVNS in the community is providing an increasing role. Thus, it is needed to describe how the home visit nursing service from HVNS has expanded and what related to the change to explore the future role of HVNS.

## Aim of this Article

In this paper we present findings and discussion of our review of Japanese psychiatric home visit nursing service from HVNS. We have examined documents, investigation about implementation rate and Survey of Medical Receipt of home visiting services from 2006 to 2016. We examined processes and outcomes of how the Japanese government has spread psychiatric home visit nursing from HVNS.

## Materials and Methods

To examine how psychiatric home visit nursing service developed, our data collection began with a search of the medical fee system of Japan during 1992 to 2016 from documents. To know the numbers of users, a survey on Home-visit Nursing Care Expenses [3] conducted by the Japanese government and held every two years were analyzed. This survey investigates the one-third of total medical receipts examined, thus we estimated the number of users of home visit nursing by tripling the reported numbers.

To calculate the implementation rate of the service, survey data conducted from 2006 to 2016 annually by some of the authors was used [4]. This data were derived from questionnaire surveys administered to all facilities belonging to The National Association for Visiting Nurse Service. The questionnaire asking ‘the presence or absence of service users who have mental disorders at that time’ were sent and returned by fax.

## Results

**Fig. 1:**
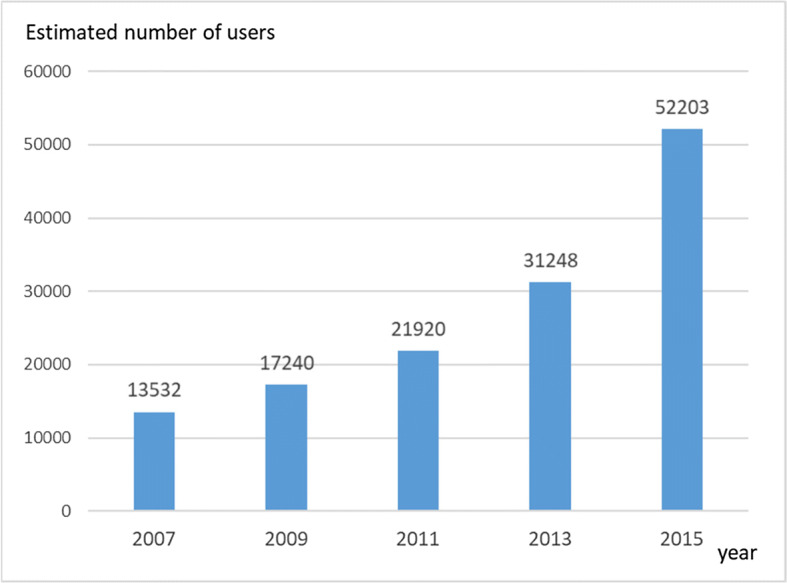
Number of clients with mental disorders receiving home visit nursing services by HVNS under medical insurance

The number of users of psychiatric home visit nursing services provided by HVNS during 2007 to 2015 increased from 13,532 to 52,203. From 2013 to 2015 there was a large increase in user numbers, from 31,248 to 52,203 (Fig. 1).

Data used in Fig. 1 is based on ‘Survey on Home-Visit Nursing Care Expenses’ by Ministry of Health, Labour and Welfare [3].

**Fig. 2:**
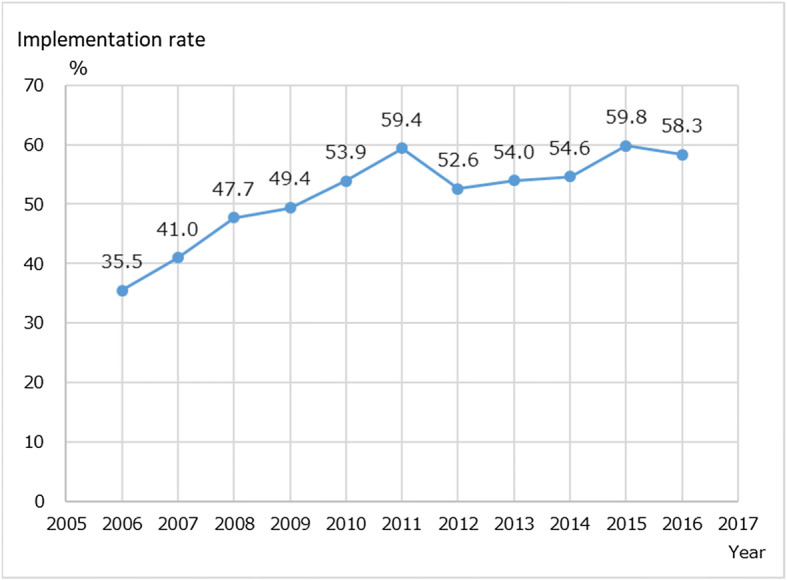
Implementation rate of Psychiatric home visit nursing among HVNS during 2006–2016

The implementation rate of psychiatric home visit nursing services among HVNS (percentage of HVNS providing home-visit nursing service to the psychiatric clients among all the survey respondent HVNS) during 2006 to 2017 also increased steadily from 35.5% in 2006 to 58.3% in 2016 (Fig. 2).

In 1992, HVNS was established only for those over 65 years old. In 1994 HVNS started to provide services through National Medical insurance (Table 1). Mental diseases were included in this, but nurses anecdotally perceived it was not safe to visit patients alone and hesitated to start visiting those patients [4]. Multiple nurses’ home visiting were not accepted by National Medical Insurance, so HVNS had to cover the fees for additional nurse’s costs during this phase. The recipients of HVNS’s home visiting was defined as “Patients with difficulties in visiting hospital due to their diseases and injuries” since 1994. Many psychiatric patients are able to go out from their home physically. They can use outpatient services, activity services by local governments and sometimes even have employment. During this phase, the numbers of patients in receipt of the service slightly increased from 13,532 in 2007 to 17,240 in 2009.

**Table 1:** Development of reimbursement system for psychiatric home visiting services

1992	Establishment of HVNS under Health and Medical Service Act for the Aged
1994	‘Home Visit Nursing’ paid by National Medical Insurance -Home visit nursing for psychiatric patients started to be reimbursed along with other physical diseases
2000	‘Long-term Care Insurance Act’ -Accelerate the establishment of HVNS
2004	‘The REFORM VISION of Mental Health and Welfare’ was released -policy from institutional care towards community care
2008	Additional payment for ‘24 h support’ -24 h telephone consultation and emergency visit if needed
2010	Additional payment for ‘Home visit nursing by multiple nurses’ -For those with violent, nuisance or destruction behavior
2012	‘Psychiatric Home Visit Nursing’ paid by National Medical Insurance -Specific payment for the clients with mental disorders in community -Care for the family members also included -Provided by the nurses with experience in psychiatry or programmed training -Ordered by psychiatrists

The REFORM VISION of Mental Health and Welfare policy was released in 2004 [2]. In this vision, approximately 70,000 patients could be discharged from hospital. The Government moved to recommend using home visiting more for psychiatric patients. Multiple nurses’ home visiting were also then covered by National Medical Insurance in 2010. As a result, the implementation rate in 2011 raised to 59.4% and the numbers in receipt of the service increased to 21,920.

In 2012, medical insurance coverage was revised in which ‘psychiatric home visit nursing’ was differentiated and redefined. In this coverage, family care and short-term visits were also included, however it was also required that HVNS meet certain conditions (i.e. Nurses with experience or training in psychiatric home visit nursing) and that patients register for the reimbursement of payment. The implementation rate declined temporarily to 52.6% (592/1125) in 2012, but increased again to 54.0% in 2013 and gradually increased to reach 58.3% (1179/2024) in 2016. The ‘psychiatric home visit nursing’ for older users (+65) who previously were covered by Long-Term Care Insurance were now covered by medical insurance since 2014. The numbers of patients in receipt of the service then increased to 31,248 in 2013 and 52,203 in 2015. Although population in Japan is slightly decreasing from the peak of 128millions in 2008 to 126.8millions in 2017, number of users constantly increase during the same period.

## Conclusions

Numbers summarized briefly in this paper demonstrate the impact of policy on psychiatric service usage in Japan. The medical insurance system has enhanced policy changes. The dissemination of psychiatric home visiting nursing is now progressing rapidly. We should be able to detect the outcome of psychiatric home visiting nursing in influencing patient’s quality of daily life. Especially after 2012, over 20,000 nurses in Japan now participate in 20 h of programmed training course every year, to assist in changing knowledge, skills and practice in assisting psychiatric patients in their own homes. If they can understand the context of psychiatric patients and their care clearly, they can be a strong ally for community inclusion. Mental health is a community-wide concern. Community psychiatric nursing is helping to deliver better quality of life and to hear the voice of mentally ill patients.
